# Pattern of long-term weight and metabolic changes after a first episode of psychosis: Results from a 10-year prospective follow-up of the PAFIP program for early intervention in psychosis cohort

**DOI:** 10.1192/j.eurpsy.2022.2308

**Published:** 2022-08-16

**Authors:** J. Vázquez-Bourgon, M. Gómez-Revuelta, J. Mayoral-van Son, J. Labad, V. Ortiz-García de la Foz, E. Setién-Suero, R. Ayesa-Arriola, D. Tordesillas-Gutiérrez, M. Juncal-Ruiz, B. Crespo-Facorro

**Affiliations:** 1 Department of Psychiatry, University Hospital Marqués de Valdecilla—Instituto de Investigación Marqués de Valdecilla (IDIVAL), Santander, Spain; 2 Centro de Investigación Biomédica en Red en Salud Mental (CIBERSAM), Seville, Spain; 3 Departamento de Medicina y Psiquiatría, Universidad de Cantabria, Santander, Spain; 4 Department of Psychiatry, School of Medicine, University Hospital Virgen del Rocio-IBiS, Seville, Spain; 5 Department of Psychiatry, Hospital de Mataró, Consorci Sanitari del Maresme, Mataró, Spain; 6 Translational Neuroscience Research Unit I3PT-INc-UAB, Institut de Innovació i Investigació Parc Taulí (I3PT), Institut de Neurociències, Universitat Autònoma de Barcelona, Barcelona, Spain; 7 Department of Psychiatry, Sierrallana Hospital—Instituto de Investigación Marqués de Valdecilla (IDIVAL), Torrelavega, Spain

**Keywords:** Cholesterol, glucose, medication-naïve, second-generation antipsychotic, triglycerides, weight gain

## Abstract

**Background:**

People with psychosis are at higher risk of cardiovascular events, partly explained by a higher predisposition to gain weight. This has been observed in studies on individuals with a first-episode psychosis (FEP) at short and long term (mainly up to 1 year) and transversally at longer term in people with chronic schizophrenia. However, there is scarcity of data regarding longer-term (above 3-year follow-up) weight progression in FEP from longitudinal studies. The aim of this study is to evaluate the longer-term (10 years) progression of weight changes and related metabolic disturbances in people with FEP.

**Methods:**

Two hundred and nine people with FEP and 57 healthy participants (controls) were evaluated at study entry and prospectively at 10-year follow-up. Anthropometric, clinical, and sociodemographic data were collected.

**Results:**

People with FEP presented a significant and rapid increase in mean body weight during the first year of treatment, followed by less pronounced but sustained weight gain over the study period (Δ15.2 kg; SD 12.3 kg). This early increment in weight predicted longer-term changes, which were significantly greater than in healthy controls (Δ2.9 kg; SD 7.3 kg). Weight gain correlated with alterations in lipid and glycemic variables, leading to clinical repercussion such as increments in the rates of obesity and metabolic disturbances. Sex differences were observed, with women presenting higher increments in body mass index than men.

**Conclusions:**

This study confirms that the first year after initiating antipsychotic treatment is the critical one for weight gain in psychosis. Besides, it provides evidence that weight gain keep progressing even in the longer term (10 years), causing relevant metabolic disturbances.

## Introduction

People with a schizophrenia spectrum disorder present an excess morbidity and mortality when compared with the general population, leading to a reduced life expectancy [1–3]. It has been described that cardiometabolic disorders are among the main natural causes of this excess mortality [4, 5]. Weight increments and related metabolic changes explaining the latter cardiometabolic events appear early in the course of the psychotic disorder [6]. In this sense, the first year of the first-episode psychosis (FEP) appears as the critical period for the development of the metabolic disturbances. For instance, up to 80% of the weight increase observed at long term (3 years) occurred in this critical period; despite of this, FEP participants continued gaining weight even after this critical period, although at a slower pace [7]. This observation is also supported by other studies showing that people with psychosis present weight gain and metabolic disturbances during the first 3–4 years of initiating the antipsychotic treatment [8, 9]. Studies, based on individuals with stablished schizophrenia, following cross-sectional and retrospective methodologies, also showed the presence of weight increments even at longer periods [10–13]. Together with this increase in body weight, and mainly as a consequence of it [7, 14], a wide range of lipid and glycemic metabolism alterations appear progressively in people with psychosis in parallel with the progression of the mental disorder. For instance, Gardner-Sood et al. [11] reported in a group of 450 participants with chronic psychosis that half of them were obese, and 57% met criteria for metabolic syndrome, while a fifth met the criteria for type 2 diabetes mellitus. Similarly, Henderson et al. [13] reported an increase in body mass index (BMI), cholesterol, triglycerides, and the risk for diabetes, in people with schizophrenia, after 10 years of having initiated treatment with clozapine. Although there is mounting evidence regarding a differential effect of antipsychotic treatments on weight gain [15], there is scarcity of evidence from prospective longitudinal studies analyzing the progression of weight and metabolic changes at long term (10 years) in people with FEP; most of the scientific evidence comes from short-term and cross-sectional studies or from retrospective reviews of medical records.

Taking into account the above evidences, we hypothesize that people with FEP will present, at 10-year follow-up, a significant increase in weight and metabolic disturbances that will be significantly greater than that observed in healthy participants. This study aims to explore prospectively the pattern of weight changes and the occurrence of metabolic disturbances in a cohort of individuals with a FEP followed during the first 10 years after the breakout of their FEP. And secondly, to compare them with a group of healthy (without psychiatric disorders) participants.

## Methods

### Study setting

The study was part of a prospective longitudinal project on FEP, the “First Episode Psychosis Clinical Program 10” (PAFIP10) study [16]. PAFIP-10 is a follow-up at approximately 10 years (range between 8 and 12) of a cohort of individuals with a first episode of nonaffective psychosis initially included in the Cantabria Program for Early Intervention in Psychosis (PAFIP) [17].

The study was approved by the local ethics committee for clinical research (CEIm Cantabria) in accordance with international standards for research ethics (trial number NCT02200588). Participants included in the study provided written informed consent for entry initially into PAFIP and for PAFIP-10 reassessment.

### Baseline inclusion criteria

All referrals to PAFIP between February 2001 and July 2008 were screened against the following inclusion criteria: age 15–60 years; living in the catchment area; experiencing a FEP; no prior treatment with antipsychotic medication or, if previously treated, a total lifetime of adequate antipsychotic treatment of less than 6 weeks. DSM-IV criteria for drug or alcohol dependence, intellectual disability, and having a history of neurological disease or head injury were exclusion criteria. The diagnoses were confirmed through the Structured Clinical Interview for DSM-IV (SCID–I) [18]. A group of subjects without psychiatric illness was recruited as control group through public advertisements, from the same catchment area. Individuals in the control group were matched to FEP cases for age, sex, and ethnicity.

### Participants’ clinical assessment

Sociodemographic and clinical information was recorded from interviews with participants, their relatives, and from medical records on each time point. Clinical data were collected at four different points throughout the 10-year period; baseline, 1-, 3- and 10-year follow-up. Clinical symptoms of psychosis were assessed by the Scale for the Assessment of Negative Symptoms (SANS) [19] and the Scale for the Assessment of Positive Symptoms (SAPS) [20], and general psychopathology with the Brief Psychiatric Rating Scale (BPRS) [21]. Participants’ weight and waist circumference were obtained at all time points, while height was measured at the time of enrollment.

Participants from the healthy control group were evaluated at baseline and 10 years after, where sociodemographic and biometric data were collected.

### Laboratory analyses

All laboratory determinations were performed at the same site, in our hospital, after an overnight fast, at baseline, 1-, 3-, and 10-year follow-ups. Fasting state, as well as treatment compliance (good compliance: taking ≥ 90% of the prescribed treatment), was reported by patients and their family members. Glucose, total cholesterol, high density lipoprotein (HDL) cholesterol, and triglycerides were measured by automated methods on a TechniconDax (Technicon Instruments Corp, Tarrytown, NY), using the reagents supplied by Boehringer-Mannheim (Mannheim, Germany). Low density lipoprotein (LDL) cholesterol was determined by the Friedewald et al. [23] calculation. Insulin levels were measured by an immunoradiometric assay (Immunotech, Beckman Coulter Company, Prague, Czech Republic), where values for normal weight subjects are 2–17 μU/ml. Insulin resistance was calculated using the homeostasis model assessment (HOMA) index, by means of a previously described formula [22], and through the triglyceride/HDL cholesterol (TG/HDL) ratio, as proposed by McLaughlin et al. [24], and with the cut-off point of 3.5.

### Statistical analyses

To evaluate the changes over time of weight, BMI, lipid, and glycemic measurements in FEP, we used repeated-measures analyses of variance (ANOVA) followed by post-hoc Bonferroni tests. For these analyses, we included baseline, 1-, 3-, and 10-year measurements.

Next, we explored if there were significant differences in the longitudinal changes between people with FEP and controls. For this, we carried out ANCOVA models where mean variable change (10-year minus baseline measures) was used as the dependent variable, subject group (FEP vs. control) as the fixed factor, and baseline BMI, baseline measurement data, age, and sex as covariates.

To evaluate the clinical impact of the metabolic changes observed in the FEP group, we additionally calculated the percentage of participants with pathologic values in BMI and glucose and lipid variables (according to the reference values of our laboratory) at baseline and at 10-year follow-up. To evaluate significant changes in these percentages, we used the McNemar test for repeated measures.

Finally, we studied the association between weight gain and metabolic changes. For this, we performed a partial correlation analysis controlling by sex, age, and baseline BMI. And subsequently, we compared the changes in glycemic and lipid measurements in three different FEP groups regarding the increase in BMI from baseline (7, 15 [25]): less than 7% increase, between 7 and 20%, and greater than 20%. The three groups were compared using covariance analysis.

Secondary analyses were carried out to explore sex effect on the long-term weight and metabolic changes in the FEP cohort through ANCOVA test. And finally, prediction factors of weight gain were explored using a cumulative odds ordinal logistic regression with proportional odds to determine the effect of sex, age, duration of untreated psychosis (DUP), treatment discontinuation, weight gained at 1 year, HOMA index, leptin, triglycerides, LDL cholesterol, and HDL cholesterol at baseline on critical weight gain (≥20% from baseline) at 10 years.

The Statistical Package for Social Science (SPSS) version 22.0 (IBM, Armonk, NY) was used for the statistical analyses. All statistical tests were two-tailed, and the significance was determined at the 0.05 level.

## Results

### Characteristics of the cohort

**Figure 1.**
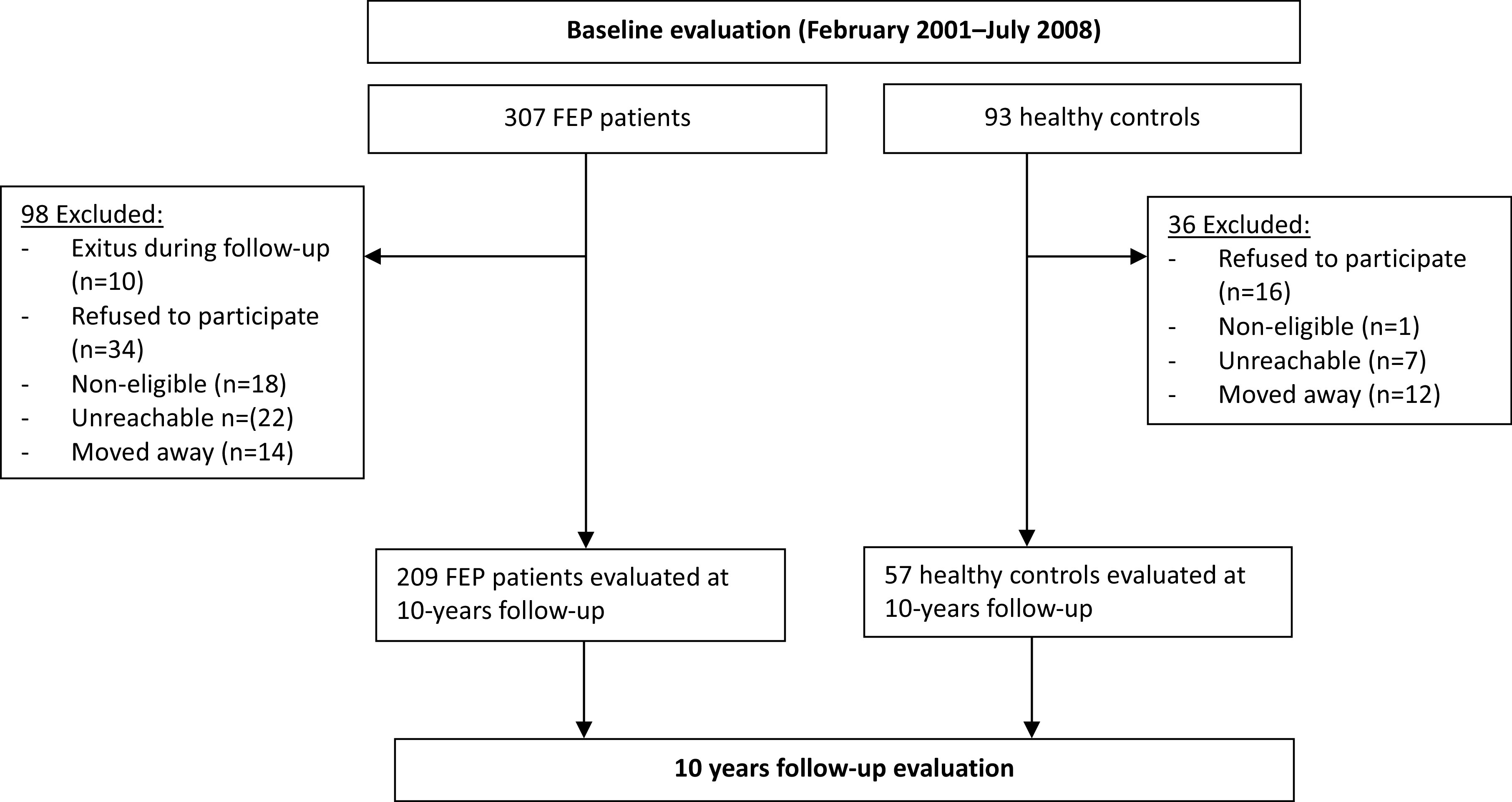
Participants’ flow in the study.

A total of 209 patients and 57 healthy controls were evaluated after 10 years of having initiated treatment (Figure 1). FEP patients and healthy controls had similar baseline sociodemographic characteristics (Table 1). At program admission FEP patients had a mean age of 29.3 years (SD = 8.8), most of them were men (54.5%), white (98.6%), from low-middle socioeconomic status (54.6%), and 71.8% lived with family. Mean DUP was 13.6 months (SD = 29.7), and at presentation the mean SAPS, SANS, and BPRS were 13.3 (SD = 4.6), 7.8 (SD = 6.4), and 62.0 (SD = 13.2), respectively. At baseline, 56.5% reported consuming tobacco, 51.7% alcohol, and 37.8% cannabis. At 6-month follow-up, 61.2% had a confirmed diagnosis of schizophrenia, 1.4% schizoaffective, 23.4% schizophreniform, 7.2% brief psychotic episode, and 6.7% unspecified psychotic disorder. At the 10-year follow-up, we evaluated 57 individuals from the control group.

**Table 1.** Sociodemographic and clinical characteristics of study population.

	FEP patients *N* = 209		Healthy controls *N* = 57		Total *N* = 266		Statistics		
	Mean	SD	Mean	SD	Mean	SD	Stat.	Value	*p*
Age at admission, years	29.3	8.8	28.6	8.3	29.2	8.7	*t*	0.563	0.574
Education, years	10.8	3.4	10.8	2.5	10.8	3.2	*t*	−0.117	0.907
	*N*	%	*N*	%	*N*	%			
Sex, male	114	54.5	33	57.9	147	55.3	*X* ^2^	0.203	0.652
Ethnicity, white	206	98.6	53	100.0	259	98.9	Fisher	0.770	1.000
Education level, secondary or lower	92	44.0	17	32.1	109	41.6	*X* ^2^	2.482	0.115
Family socioeconomic status, not/low qualified	113	54.6	20	38.5	133	51.4	*X* ^2^	4.327	0.038
Single	158	75.6	13	50.0	171	72.8	*X* ^2^	7.646	0.006
Employed/Studying, yes	115	55.0	23	88.5	138	58.7	*X* ^2^	10.666	0.001
Tobacco smoking, yes	118	56.5	31	55.4	149	56.2	*X* ^2^	0.022	0.883
Cannabis use, yes	79	37.8	16	28.6	95	35.8	*X* ^2^	1.635	0.201
Alcohol drinking, yes	108	51.7	33	62.3	141	53.8	*X* ^2^	1.908	0.167

Abbreviation: FEP, first-episode psychosis.

The majority of patients (95.2%, *n* = 199) in this sample were antipsychotic-naïve at study entry. The other 10 patients (4.8%) had been treated with antipsychotics prior to their inclusion in the study, although during a short period of time (median = 5 days). According to the patients and their families’ reports on treatment compliance, 76.9% of patients were good compliers at 1 year, 79.1% at 3 years, and 92.7% at 10 years. Some of the patients were on other concomitant psychopharmacological treatments that potentially contribute to weight gain. Thus, 38 patients (18.2%) were on antidepressant treatment at 1-year follow up, 30 (14.3%) at 3 years, and 36 (17.2%) at 10 years. Besides, six patients (2.9%) were on treatment with mood stabilizers at 1-year follow-up, 13 (6.2%) at 3 years, and 29 (13.9%) at 10 years. Patients presented a high rate of antipsychotic treatment switch during the study period (Supplementary Table S3).

### Weight and metabolic differences after 10 years of psychosis breakout

Individuals with FEP presented a significant increase in their mean weight and BMI, 10 years after their psychosis diagnosis. The mean weight gain in this period was 15.2 kg (SD = 12.6) (Table 2). The majority (80.8%) of patients experienced a clinically significant weight gain (>7% from baseline), with 32.5% of patients having increased between 20 and 40% of their basal body weight, and 18.7% even above 40% of their basal weight.

**Table 2.** Descriptive data and ANOVA repeated measures analyses of body weight and metabolic changes during the first 10 years of antipsychotic treatment in a population of individuals with a first-episode psychosis.

	Baseline mean (SD)	*n*	1 year mean (SD)	*n*	3 years mean (SD)	*n*	10 years mean (SD)	*n*	F repeated measures^a^	df	*p*	Partial eta squared
*Anthropometric changes*												
Weight (kg)	67.0 (13.4)	206	76.0 (15.5)	197	77.4 (16.3)	187	82.4 (18.3)	206	103.9	3; 176	<0.001	0.643
BMI (kg/m^2^)	23.6 (3.7)	204	26.6 (4.3)	195	27.3 (4.7)	186	29.0 (5.8)	204	105.4	3; 176	<0.001	0.646
*Lipid parameters*												
Cholesterol (mg/dl)	174.6 (36.9)	207	193.9 (38.6)	199	195.7 (40.1)	193	197.3 (38.6)	199	26.2	3; 178	<0.001	0.310
LDL cholesterol (mg/dl)	108.5 (32.7)	183	123.5 (33.1)	196	120.7 (33.1)	188	122.5 (32.2)	191	17.3	3; 148	<0.001	0.263
HDL cholesterol (mg/dl)	50.4 (13.9)	182	49.8 (12.4)	196	51.0 (14.7)	191	28.9 (13.1)	194	2.2	3; 151	0.087	0.043
Triglycerides (mg/dl)	84.4 (39.9)	181	114.6 (87.2)	195	120.6 (115.8)	192	129.7 (80.4)	195	20.7	3; 151	<0.001	0.295
*Glycemic parameters*												
Glucose (mg/dl)	88.0 (21.0)	205	88.7 (13.7)	199	89.9 (13.7)	193	92.3 (14.1)	200	2.9	3; 177	0.037	0.048
HOMA index	1.9 (2.2)	157	2.2 (2.2)	170	2.6 (2.2)	184	3.0 (2.3)	177	6.7	3; 97	<0.001	0.177
HOMA index, men	1.7 (1.5)	85	2.3 (2.6)	94	2.8 (2.6)	99	3.3 (2.7)	97	5.1	3; 51	0.004	0.237
HOMA index, women	2.2 (2.7)	72	2.1 (1.7)	76	2.3 (1.7)	85	2.8 (1.7)	80	3.5	3; 45	0.023	0.201
Triglyceride/HDLc index	1.8 (1.1)	177	2.6 (2.6)	195	2.7 (2.7)	191	3.0 (2.3)	194	17.6	3; 146	<0.001	0.269
Insulin total (μU/ml)	9.1 (9.3)	159	9.9 (8.6)	170	11.2 (7.7)	185	12.8 (8.2)	177	5.2	3; 100	0.002	0.139
Insulin men	8.2 (7.0)	85	9.9 (9.6)	94	11.8 (8.7)	100	13.4 (9.5)	97	4.5	3; 53	0.007	0.213
Insulin women	10.1 (11.4)	74	9.9 (7.2)	76	10.5 (6.4)	85	12.1 (6.2)	80	3.3	3; 47	0.030	0.183
*Hormonal levels*												
Leptin total (ng/ml)	8.4 (8.3)	153	13.2 (10.5)	184	13.3 (11.8)	184	21.5 (18.5)	174	35.5	3; 106	<0.001	0.508
Leptin men	4.8 (5.0)	84	8.2 (6.6)	104	8.6 (8.4)	98	13.2 (11.4)	94	17.8	3; 58	<0.001	0.492
Leptin women	12.7 (9.3)	69	19.8 (11.1)	80	18.6 (12.9)	86	31.3 (20.5)	80	21.9	3; 48	<0.001	0.595

Abbreviations: BMI, body mass index; HDL, high density lipoprotein; HOMA, homeostasis model assessment; LDL, low density lipoprotein.

a Pillai’s trace statistic *F* value.

These increments in body weight had a relevant clinical effect; the percentage of obese (BMI ≥ 30 kg/m^2^) among the individuals with psychosis rose from 7 to 36.8% at the end of the 10-year follow-up. The percentage of those meeting criteria for overweight (BMI ≥ 25, <30 kg/m^2^) also increased, from 23.9 to 36.3%, in the same period, leaving just a mere 26.5% of individuals with normal weight at 10-year follow-up (from 69.9% at baseline).

The maximum increase in weight occurred in the first year of treatment; 58.4% of the total increment in mean weight and 55.6% of the total increment in mean BMI (see Table 2) occurred in the first year. From year 1 to year 3, the weight gain decreased (trend toward statistical significance), reaching again statistical significance in the last 7 years of study period. These data showed a sustained increase in mean weight and BMI following the first year of treatment, with an average increase of 0.7 kg each year (0.35 kg/m^2^ per year). Leptin plasma levels, as an indicator of adipose mass, followed a similar trajectory to weight change.

Similarly, all lipid and glucose parameters showed a significant increase during the 10-year follow-up. Only HDL cholesterol levels showed a statistical tendency (*p* = 0.087) to decrease from baseline to the end of the study period (Table 2).

### Longitudinal differences in weight and metabolism changes between people with psychosis and healthy controls

Healthy control individuals also present significant increments in mean body weight at 10-year evaluation (Supplementary Table S2). However, when comparing people with psychosis and healthy controls, we observed that over the 10-year follow-up period, the individuals with psychosis presented a significantly worse progression in weight and metabolic measurements (Table 3). For instance, the psychosis group showed a greater increase in mean weight (15.21 vs. 2.89 kg, *F* = 44.58, *p* < 0.001) and BMI (5.41 vs. 1.03, *F* = 42.89, *p* < 0.001) than the control one.

**Table 3.** Differences in longitudinal changes in anthropometric and metabolic measurements between individuals with psychosis and healthy controls after 10 years of follow-up.

	People with FEP	Healthy controls	Stats^a^		
	Mean diff. (SD)	Mean diff. (SD)	df	*F*	*p*
*Anthropometric measures*					
Weight (kg)	15.21 (12.63)	2.89 (7.35)	1; 254	45.61	<0.001
BMI (kg/m^2^)	5.41 (4.55)	1.03 (2.74)	1; 254	43.40	<0.001
Waist circumference (cm)	13.63 (11.74)	4.68 (7.14)	1; 86	16.51	<0.001
*Lipid parameters*					
Cholesterol (mg/dl)	21.64 (33.95)	8.79 (36.52)	1; 217	1.42	0.235
HDL (mg/dl)	−1.56 (12.49)	2.75 (6.86)	1; 189	6.51	0.012
LDL (mg/dl)	13.80 (28.16)	6.54 (31.45)	1; 186	0.54	0.464
Triglycerides (mg/dl)	44.45 (72.75)	−2.75 (52.79)	1; 189	9.53	0.002
*Glycemic parameters*					
Glucose (mg/dl)	4.15 (21.68)	4.42 (16.85)	1; 216	4.48	0.036
HOMA index	1.07 (3.19)	0.16 (0.87)	1; 152	13.12	<0.001
HOMA index, men	1.54 (2.79)	−0.19 (0.93)	1; 80	6.54	0.013
HOMA index, women	0.54 (3.53)	0.51 (0.67)	1; 72	7.907	0.006
Triglycerides-HDL index	1.13 (2.06)	−0.07 (0.97)	1; 185	8.49	0.004
Insulin (μU/ml)	3.64 (12.29)	0.19 (3.59)	1; 155	15.609	<0.001
Insulin, men	5.15 (10.20)	−0.78 (3.72)	1; 81	8.36	0.005
Insulin, women	1.99 (14.14)	1.25 (3.27)	1; 74	8.64	0.004
*Hormonal levels*					
Leptin (ng/ml)	12.42 (14.55)	4.66 (4.52)	1; 150	9.92	0.002
Leptin, men	7.78 (10.51)	3.85 (4.65)	1; 78	3.49	0.066
Leptin, women	17.45 (16.59)	5.54 (4.39)	1; 72	8.90	0.004

Abbreviations: BMI, body mass index; BP, blood pressure, HDL, high density lipoprotein; HOMA, homeostasis model assessment; LDL, low density lipoprotein.

a Statistics: ANCOVA model: parameter change was used as the dependent variable, participants group (psychosis vs. controls) was the fixed factor and baseline BMI, baseline parameter data, age, and sex were used as covariates.

### Incidence of clinically relevant abnormal metabolic measurements after 10 years of psychosis breakout

The percentage of individuals with psychosis meeting criteria for hypertriglyceridemia (>150 mg/dl) and hypercholesterolemia (total cholesterol > 200 mg/dl) increased significantly, from 7.1 to 24.4% (*p* < 0.001) and from 23.4 to 41.6% (*p* < 0.001), respectively, during the 10-year study period (Table 4). In the same line, the proportion of FEP patients with clinically elevated LDL cholesterol (>130 mg/dl) rose from 24.2 to 35.2% (*p* = 0.006). However, no significant changes were observed in the percentage of FEP patients with low HDL cholesterol (Table 4).

**Table 4.** Comparison of proportion of FEP participants with pathologic parameters in weight, glycemic, and lipid parameters at baseline and at 10-year follow-up.

	10 years follow-up	Baseline			
	% (*n*)	% (*n*)	% difference	N	*p* ^a^
*Anthropometric changes*					
Obesity, BMI ≥ 30 kg/m^2^	36.8 (74)	7.0 (14)	29.8	201	<0.001
Overweight, BMI ≥ 25,<30 kg/m^2^	36.3 (73)	23.9 (48)	12.4	201	0.011
*Glycemic parameters*					
Glucose > 110 mg/dl	7.1 (14)	1.5 (3)	5.6	196	0.007
Insulin total (μU/ml)	31.3 (41)	9.2 (12)	22.1	131	<0.001
Levels in men > 15.7	36.2 (25)	7.2 (5)	29.0	69	<0.001
Levels in women > 17.3	25.8 (16)	11.3 (7)	14.5	62	0.078
HOMA index	30.2 (39)	7.0 (9)	23.2	129	<0.001
Levels in men > 3.5	37.7 (26)	2.9 (2)	34.8	69	<0.001
Levels in women > 3.9	21.7 (13)	11.7 (7)	10.0	60	0.238
Triglyceride/HDL index > 3.5	23.8 (39)	11.0 (18)	12.8	164	<0.001
*Lipid parameters*					
Cholesterol > 200 mg/dl	41.6 (82)	23.4 (46)	18.2	197	<0.001
LDL cholesterol > 130 mg/dl	35.2 (58)	24.2 (40)	10.8	165	0.006
HDL cholesterol < 40 mg/dl	26.2 (44)	21.4 (36)	4.8	168	0.280
Triglycerides > 150 mg/dl	24.4 (41)	7.1 (12)	17.3	168	<0.001

Abbreviations: BMI, body mass index; HDL, high density lipoprotein; HOMA, homeostasis model assessment; LDL, low density lipoprotein.

^a^ McNemar test for repeated measures.

Significant increments were also observed in the percentage of people with psychosis meeting clinically elevated levels of glucose and insulin, and both HOMA index and TG/HDL ratio, indicating a greater risk for the development of glucose metabolism alterations such as glucose intolerance.

### Correlation between long-term weight gain and metabolic changes in FEP

Weight gain was positively correlated with insulin increase (*r* = 0.48, *p* < 0.001) and with the insulin resistance indexes, HOMA (*r* = 0.48, *p* < 0.001) and triglycerides/HDL (*r* = 0.51, *p* < 0.001). A trend toward significance was observed between weight and glucose changes (*r* = 0.18, *p* = 0.055). There was also a positive correlation between weight gain and triglycerides increase (*r* = 0.46, *p* < 0.001), and a negative correlation between weight gain and HDL changes (*r* = −0.32, *p* = 0.001). These associations were significant after controlling for sex, age, and baseline BMI.

A relationship between weight increase and changes in metabolic variables was detected when we compared three groups of individuals with psychosis according to the increase in their BMI (Table 5), where significant differences were observed for insulin, HOMA index and TG/HDL ratio, and triglycerides increments, as well as for HDL cholesterol reductions, between groups.

**Table 5.** Changes in glycemic and lipid parameters after 10 years of antipsychotic treatment: comparison of groups with different percentage of weight gain.

	BMI changes (% from baseline) during the 10 year follow-up period								
	<7% BMI increase from baseline	7–19.9% BMI increase from baseline	≥20% BMI increase from baseline						
	Mean (IC95%)	Mean (IC95%)	Mean (IC95%)					*p* ^b^	
	A: *N* = 39	B: *N* = 60	C: *N* = 104	df	*F* ^a^	*p*	A versus B	A versus C	B versus C
*Change in glucose parameters*									
Glucose (mg/dl)	1.2 (−6.1, 8.5)	3.9 (−1.8, 9.6)	5.3 (0.9, 9.8)	2; 190	0.46	0.633	1.000	1.000	1.000
Insulin total (μU/ml)	0.3 (−4.2, 4.8)	−1.6 (−5.3, 2.1)	8.4 (1.5, 11.3)	2; 129	9.94	<0.001	1.000	0.012	<0.001
HOMA index	−0.1 (−1.2, 1.1)	−0.2 (−1.1, 0.7)	2.3 (1.6, 3.1)	2; 127	10.43	<0.001	1.000	0.003	<0.001
Triglyceride/HDL index	0.4 (−0.3, 1.1)	0.2 (−0.4, 0.7)	2.0 (1.6, 2.4)	2; 159	16.10	<0.001	1.000	<0.001	<0.001
*Change in lipid parameters*									
Cholesterol (mg/dl)	12.4 (1.1, 23.7)	23.3 (14.3, 32.3)	24.2 (17.3, 31.2)	2; 191	1.62	0.200	0.408	0.251	1.000
LDL cholesterol (mg/dl)	6.8 (−3.7, 17.3)	14.0 (5.9, 22.2)	16.2 (9.9, 22.5)	2; 160	1.11	0.330	0.850	0.415	1.000
HDL cholesterol (mg/dl)	1.1 (−3.3, 5.4)	3.6 (0.2, 7.0)	−5.4 (−8.0, −2.8)	2; 163	9.20	<0.001	1.000	0.040	<0.001
Triglycerides (mg/dl)	17.5 (−6.7, 41.7)	14.7 (−4.9, 34.3)	72.3 (57.3, 87.3)	2; 163	13.16	<0.001	1.000	0.001	<0.001

Abbreviations: BMI, body mass index; HDL, high density lipoprotein; HOMA, homeostasis model assessment; LDL, low density lipoprotein.

a ANCOVA model: parameter change was used as the dependent variable, weight gain group was the fixed factor, and baseline BMI, age, and sex were used as covariates.

b Pairwise comparisons based on estimated marginal means; Bonferroni adjustment for multiple comparisons.

### Secondary analyses: sex differences in weight and metabolic changes in individuals with FEP at longer term (10 years)

Women with psychosis presented a significantly greater increment in BMI (6 vs. 4.9, *F* = 4.18, *p* = 0.042) and leptin levels (17.45 vs. 7.78, *F* = 23.29, *p* < 0.001) than men (Supplementary Table S1). On the contrary, men presented a worse evolution in glucose (5.65 vs. 2.32, *F* = 8.00, *p* = 0.005) and HDL measurements (−1.77 vs. −1.32, *F* = 4.01, *p* = 0.045) than women. No other significant differences were observed in long-term metabolic variables changes regarding sex.

### Secondary analyses: prediction factors of weight increase at 10-year follow-up

A cumulative odds ordinal logistic regression with proportional odds was run to determine the effect of sex, age, DUP, treatment discontinuation, weight gained at 1 year, HOMA index, leptin, triglycerides, LDL cholesterol, and HDL cholesterol at baseline on weight gain at 10 years.

According to the logistic regression results, the assumption of proportional odds was met, as assessed by a full likelihood ratio test comparing the fit of the proportional odds model to a model with varying location parameters, χ^2^(8) = 10.079, *p* = 0.344. The Pearson goodness-of-fit test indicated that the model was a good fit to the observed data (χ^2^(226) = 247,756, *p* = 1.096).

Among the variables within the model, DUP had a statistically significant effect on the prediction of whether there is a weight increase greater than 20% (Wald *χ* ^2^(1) = 4.928, *p* = 0.026). In the same direction, the first year weight increase percentage had a statistically significant effect on the prediction of whether there is a weight increase greater than 20% at 10 years (Wald *χ* ^2^(1) = 16.189, *p* < 0.001). And the odds ratio of being in a higher category of the dependent variable (weight increase ≥ 20%) for men versus women was 3.178 (95% CI, 1.137–8.85), a statistically significant effect (*χ* ^2^(1) =4.858, *p* = 0.028). On the contrary, the odds of people with psychosis, having discontinued antipsychotic treatment, to have a weight increase greater than 20% at 10 years was 0.266 (95% CI, 0.096–0.737) times that those that continued taking antipsychotics, a statistically significant effect (*χ* ^2^(1) = 6.473, *p* = 0.011).

## Discussion

### Long-term weight differences

This study provides evidence, through a prospective methodology, of a significant increment in body weight and BMI at longer term (10 years) in people with FEP. Thus, the participants with FEP in this study presented, after their first 10 years of follow-up, an average increment of 15 kg in body weight. These results are in line with previous prospective studies on FEPs patients [6, 7, 14, 26] reporting continuous increments in mean body weight and BMI at long term (1 and 3 years). Based mainly on indirect data from transversal and retrospective studies on patients with chronic psychosis [8, 10–13], we can determine that this weight increment is also present after longer periods of antipsychotic exposure (10 years). Besides, a unique prospective study on the weight progression at longer periods [27], describing the 20-year BMI progression of 146 patients after their first hospital admission due to psychosis, reported a high prevalence of obesity among patients with schizophrenia at longer term, reaching 50% at 10 years and 62.1% at the 20-year follow-up.

Interestingly, we observed significant differences in the weight change presented at 10-year follow-up between FEP patients and healthy controls; participants in the control group also presented significant increments in body weight (Δ3.4 kg) and BMI (Δ1 kg/m^2^), but these were significantly smaller than that observed in the psychosis group. Moreover, the rate of obesity among the control group at 10-year follow-up (15.8%) was comparable to the prevalence in the Spanish general population (ranging from 14.5 to 18.0%) [28]. In contrast, the rate of obesity among the FEP group at 10-year follow-up was 36.8%, clearly above that observed in the control group and in the Spanish general population.

### Clinical impact of developing obesity

Developing obesity is in itself of clinical relevance. Obese individuals present a higher all-cause excess mortality, compared with people with normal weight [29]. Moreover, presenting obesity, even without other metabolic disorders, increases the odds for a negative prognosis; metabolically healthy obese individuals are at higher risk of developing metabolic risk factors and events than nonobese persons [30]. In relation with these and previous studies [6, 7, 14], we observed that gaining weight correlated with undesirable changes in lipid and glycemic measurements in FEP patients. At long term, the progressive worsening in the biochemical levels had a clinical manifestation in the significant increase in the percentage of participants meeting criteria for obesity and metabolic disorders.

Our results showed a significant increment, in the 10-year follow-up, in the proportion of FEP patients presenting abnormal glucose metabolism measurements, including insulin resistance indexes, glucose, and insulin levels. Moreover, the mean increments after 10 years in HOMA index, TG/HDL ratio, and insulin level were significantly greater among FEP patients than in healthy controls. This poorer outcome in glucose metabolism among patients with psychosis could be in part explained by the antipsychotic exposure and other risk factors usually present in this vulnerable population. However, previous studies have suggested that individuals with psychosis may be at greater risk of presenting abnormal glucose metabolism even before the onset of psychosis [31, 32]. This probable inherent risk could be due to genetic differences; thus, it has been described an association between insulin resistance and polygenic risk score in antipsychotic naive patients [33] and other biomarkers [34], and insulin resistance in unaffected siblings of psychosis patients [35].

Despite of this and the results regarding abnormal glucose metabolism in our sample, only five individuals had a recorded diagnosis of type II diabetes mellitus at the 10-year follow-up and were prescribed oral anti-diabetic drugs. Similarly, only six persons among the psychosis group had a diagnosis of dyslipidemia and were on pharmacological treatment.

### Importance of rapid developing weight gain in psychosis

The study shows that, according to previous reports [7, 36], the first year after initiating the antipsychotic treatment is the critical one, in which most of the weight gain occurs; up to 60% of the total weight gain observed at 10 years, occurred in the first year. People with psychosis in our study presented a rapid increase in body weight during the first year of treatment, which was followed by a slower but constant increase of body weight throughout the 10-year follow-up. This initial and rapid increase in body weight during the first year predicted a critical weight gain (greater than 20% from baseline) after 10 years of follow-up. This association is in line with previous studies; Vandenberghe et al. [37, 38] reported that early antipsychotic-induced weight changes predict long-term weight gain, both in adults and adolescents with psychiatric conditions. Besides, both rapid weight gain and steady increase trajectories, such as the pattern described in our study, have been associated with higher risk of mortality. Thus, weight stability has been associated with a lower mortality [39, 40], while steady weight gainers presented a higher mortality [39, 40]. Besides, obese patients with trajectories of weight increments were at higher risk of excess mortality [42]. These evidences highlight the relevancy of BMI trajectories versus status.

### Sex differences in weight changes

We identified sex differences, where women presented a significantly greater increase in BMI and leptin levels than men, after 10-year follow-up. Sex differences in the metabolic effect of antipsychotics have been previously described [43, 44], usually indicating a greater risk of weight gain for women. Several studies carried out transversally in patients with chronic schizophrenia and long-term exposure to antipsychotic medication showed that women presented greater weight and BMI and higher rates of obesity [43, 45–46], as well as worse measures of abdominal obesity (i.e., waist circumference and waist-hip ratio) [46], than men. However, these sex differences in antipsychotic-induced weight gain have not been consistently reported in people with FEP, with contrary results at long term [14, 27, 36]. It has been suggested that these contrary results may be due to specificity to antipsychotic treatments [14, 48].

Besides, our results showed that men were at higher risk than women of presenting high increase of body weight (≥20% from baseline) at 10-year follow-up. Other variables predicting this high increase in body weight were a higher weight gain during the first year after the FEP and antipsychotic treatment continuation, in line with previous studies by our group [7, 49]. However, a longer DUP also predicted a greater weight increase at long term. DUP has been widely associated with a greater severity of psychotic symptoms at presentation [50] and to poorer outcomes of psychosis [51], and long DUP correlates with long duration of prodromal symptoms and with poorer social support and functioning [52]. In this sense, a long DUP could indirectly determine difficulties in self-care and in the access to professional care, which could ultimately lead to poorer physical health conditions, such as metabolic syndrome [53] or glucose intolerance [31], described at baseline in drug-naïve FEP patients. However, this increased risk of metabolic alterations in drug-naïve FEP patients could not be attributed to the DUP [54]. On the other hand, no previous studies have explored the effect of DUP on weight changes in FEP, limiting the possibility of comparing our results to previous literature.

The study presents several limitations. The results may be affected by sample selection bias considering recruitment was limited to individuals agreeing to participate after having been re-contacted for a 10-year follow-up. A small group of participants (patients *n* = 5, 2.4%; controls *n* = 3, 5.3%) were under 18 at study entry, and they had probably grown and got taller during the first 3 years of the study. Unfortunately, we do not have a second measure of height within the first 3 years of study, and therefore we have used their baseline height for the 1-year and 3-year follow-ups BMI calculation (Table 2). Although these individuals present small mean height increment after 10 years (from baseline 170.8 cm (SD 7.6) to 10 year 172.9 cm (SD 7.1)), this could have partially affected the results of BMI change (Table 2). Patients were not evaluated for research purposes between year 3 and year 10, thus leading to a widely spaced follow-up interval with a lack of clinical, biometric and social information, which precluded proper analyses of weight trajectories. Data were not available on some relevant variables that affect weight (e.g., diet and physical activity) [55, 56]. Dietary and healthy lifestyle counseling was delivered as clinical routine following clinicians’ discretion but was not recorded for research purposes. The clinical repercussion related to weight gain, such as glucose intolerance, type 2 diabetes, or dyslipidemia, was not rigorously explored in our study since the diagnosis of these conditions requires at least two separate abnormal measurements [57, 58]. Finally, instead of stratifying patients by weight gain over time, future works should use cardiometabolic risk calculations, as the proposed by Perry et al. [59] for FEP.

The main strength of this study is its design; a prospective longitudinal study with an uncommon long-term follow-up (10 years), on a cohort of well-characterized individuals with FEP, and its comparison with a prospective group of healthy subjects. Besides, studying a cohort of drug-naïve (at baseline) patients with a FEP facilitates avoiding the confounding effect of chronicity and previous exposure to medications with a probable effect on metabolism.

In summary, people with a FEP experienced, at long term (10 years), a significantly greater increase in BMI than healthy controls. Significant differences were also observed in mean changes in metabolic measurements, leading to significant increments in the proportion of FEP participants with obesity and metabolic disturbances. Participants gained most of the weight in the first year after their FEP, although they continued gaining weight steadily throughout the 10-year follow-up.

## Data Availability

Raw data of this study are accessible and have been deposited in a publicly available repository (Mendeley Data: http://doi.org/10.17632/b2h5gr9m3c.1).

## Supplementary material

For supplementary material accompanying this paper visit https://doi.org/10.1192/j.eurpsy.2022.2308. click here to view supplementary material
