# Supplementary material for: Pattern of long-term weight and metabolic changes after a first episode of psychosis: Results from a 10-year prospective follow-up of the PAFIP program for early intervention in psychosis cohort
Source: Eur Psychiatry. 2022 Aug 16;65(1):e48. doi: 10.1192/j.eurpsy.2022.2308 (PMC9486831; doi:10.1192/j.eurpsy.2022.2308)
Supplement: Supplementary file 1 [file S0924933822023082sup001.docx]

Table S1. Sex differences in longitudinal changes in anthropometric and metabolic measurements in individual with psychosis after 10 years of follow-up.

|  | Men | Women | Stats† |  |  |
| --- | --- | --- | --- | --- | --- |
|  | Mean diff (SD) | Mean diff (SD) | *df* | *F* | *p* |
| ***Anthropometric measures*** |  |  |  |  |  |
| Weight (kg) | 14.95 (12.59) | 15.52 (12.75) | 1; 201 | 0.552 | 0.458 |
| BMI (kg/m^2^) | 4.92 (4.15) | 6.00 (4.94) | 1; 201 | 4.18 | 0.042 |
| Waist circumference (cm) | 12.09 (12.70) | 15.17 (10.66) | 1; 64 | 0.003 | 0.953 |
| ***Lipid parameters*** |  |  |  |  |  |
| Cholesterol (mg/dl) | 20.77 (33.47) | 22.68 (34.67) | 1; 193 | 0.13 | 0.722 |
| HDL (mg/dl) | -1.77 (10.54) | -1.32 (14.51) | 1; 165 | 4.01 | 0.045 |
| LDL (mg/dl) | 12.04 (27.72) | 15.89 (28.71) | 1; 162 | 0.02 | 0.890 |
| Triglycerides (mg/dl) | 40.91 (74.14) | 48.39 (71.45) | 1; 165 | 0.039 | 0.843 |
| ***Glycaemic parameters*** |  |  |  |  |  |
| Glucose (mg/dl) | 5.65 (25.74) | 2.32 (15.39) | 1; 192 | 8.00 | 0.005 |
| HOMA index | 1.55 (2.79) | 0.54 (3.53) | 1; 128 | 0.45 | 0.502 |
| Triglycerides-HDL index | 1.17 (2.10) | 1.08 (2.01) | 1; 161 | 0.25 | 0.618 |
| Insulin (μU/ml) | 5.15 (10.20) | 1.99 (14.14) | 1; 130 | 0.003 | 0.954 |
| ***Hormonal levels*** |  |  |  |  |  |
| Leptin (ng/ml) | 7.78 (10.51) | 17.45 (16.59) | 1; 125 | 23.29 | <0.001 |

† Statistics: ANCOVA model: parameter change was used as the dependent variable, subject group (male vs female) was the fixed factor and baseline BMI, baseline parameter data and age were used as covariates.
Abbreviations: BMI=body mass index, BP=blood pressure, HOMA=homeostasis model assessment, LDL=low density lipoprotein, HDL=high density lipoprotein.

Table S2a. Changes in weight, and in glycaemic and lipid parameters after 10 years, in healthy (without psychiatric disorders) participants.

|  | 10-year  Mean (SD) | Baseline  Mean (SD) | change | *N* | *t* | *p*† |
| --- | --- | --- | --- | --- | --- | --- |
| ***Anthropometric changes*** |  |  |  |  |  |  |
| Weight (kg) | 73.9 (13.6) | 70.5 (12.5) | 3.4 | 57 | 3.415 | 0.001 |
| BMI (kg/m^2^) | 25.4 (4.3) | 24.4 (3.8) | 1.0 | 53 | 2.731 | 0.009 |
| Waist circumference (cm) | 86.8 (11.6) | 82.1 (9.9) | 4.7 | 22 | 3.075 | 0.006 |
| ***Lipid parameters*** |  |  |  |  |  |  |
| Cholesterol (mg/dl) | 197.2 (37.9) | 188.4 (37.9) | 8.8 | 24 | 1.179 | 0.250 |
| LDL cholesterol (mg/dl) | 122.9 (30.8) | 116.4 (31.1) | 6.5 | 24 | 1.019 | 0.319 |
| HDL cholesterol (mg/dl) | 56.4 (13.9) | 53.6 (12.4) | 2.8 | 24 | 1.964 | 0.062 |
| Triglycerides (mg/dl) | 89.7 (61.4) | 92.4 (40.5) | -2.7 | 24 | -0.255 | 0.801 |
| ***Glycaemic parameters*** |  |  |  |  |  |  |
| Glucose (mg/dl) | 87.7 (9.6) | 83.3 (18.9) | 4.4 | 24 | 1.284 | 0.212 |
| HOMA index | 1.64 (0.65) | 1.48 (0.69) | 0.16 | 24 | 0.908 | 0.373 |
| HOMA index, men | 1.52 (0.55) | 1.71 (0.22) | 0.19 | 12 | 0.705 | 0.495 |
| HOMA index, women | 1.76 (0.74) | 1.25 (0.55) | 0.51 | 12 | 2.635 | 0.023 |
| Triglyceride/HDLc index | 1.78 (1.60) | 1.85 (1.48) | -0.07 | 24 | -0.355 | 0.726 |
| Insulin total (μU/ml) | 7.4 (2.6) | 7.2 (2.8) | 0.2 | 25 | 0.267 | 0.792 |
| Insulin men | 6.6 (2.2) | 7.4 (3.2) | 0.8 | 13 | 0.760 | 0.462 |
| Insulin women | 8.2 (2.8) | 6.9 (2.3) | 1.25 | 12 | 1.324 | 0.212 |
| ***Hormonal levels*** |  |  |  |  |  |  |
| Leptin (ng/ml) | 13.4 (9.4) | 8.7 (7.5) | 4.7 | 25 | 5.159 | <0.001 |
| Leptin men | 7.5 (5.9) | 3.7 (2.3) | 3.8 | 13 | 2.985 | 0.011 |
| Leptin women | 19.7 (8.4) | 14.2 (7.4) | 5.5 | 12 | 4.368 | 0.001 |

†: paired t-test comparison.

Abbreviations: BMI= body mass index, HOMA=homeostasis model assessment, LDL=low density lipoprotein, HDL=high density lipoprotein.

Table S2b. Changes in weight, and in glycaemic and lipid parameters after 10 years of the first episode of psychosis

|  | 10-year  Mean (SD) | Baseline  Mean (SD) | change | *N* | *t* | *p*† |
| --- | --- | --- | --- | --- | --- | --- |
| ***Anthropometric changes*** |  |  |  |  |  |  |
| Weight (kg) | 82.4 (18.4) | 67.2 (13.4) | 15.2 | 203 | -17.19 | < 0.001 |
| BMI (kg/m^2^) | 29.0 (5.9) | 23.6 (3.7) | 5.4 | 201 | -16.88 | < 0.001 |
| ***Lipid parameters*** |  |  |  |  |  |  |
| Cholesterol (mg/dl) | 197.0 (38.2) | 175.2 (37.5) | 21.8 | 197 | -9.0 | < 0.001 |
| LDL cholesterol (mg/dl) | 121.5 (31.8) | 107.7 (30.5) | 13.8 | 165 | -6.3 | < 0.001 |
| HDL cholesterol (mg/dl) | 48.7 (13.2) | 50.5 (13.9) | 1.7 | 168 | 1.8 | 0.075 |
| Triglycerides (mg/dl) | 129.4 (79.3) | 84.2 (40.5) | 45.2 | 168 | -8.0 | < 0.001 |
| ***Glycaemic parameters*** |  |  |  |  |  |  |
| Glucose (mg/dl) | 92.4 (14.2) | 88.2 (21.4) | 4.2 | 196 | -2.75 | 0.006 |
| HOMA index | 3.2 (2.3) | 2.1 (2.3) | 1.1 | 129 | -3.89 | < 0.001 |
| HOMA index, men | 3.4 (2.6) | 1.8 (1.6) | 1.6 | 69 | -4.70 | < 0.001 |
| HOMA index, women | 2.9 (1.8) | 2.3 (2.9) | 0.6 | 60 | -1.18 | 0.241 |
| Triglyceride/HDLc index | 3.0 (2.3) | 1.9 (1.2) | 1.2 | 164 | -7.20 | < 0.001 |
| Insulin total (μU/ml) | 13.2 (7.9) | 9.4 (9.8) | 3.7 | 131 | -3.46 | 0.001 |
| Insulin men | 13.7 (8.9) | 8.4 (7.6) | 5.3 | 69 | -4.30 | < 0.001 |
| Insulin women | 12.6 (6.6) | 10.6 (11.8) | 2.0 | 62 | -1.11 | 0.272 |
| ***Hormonal levels*** |  |  |  |  |  |  |
| Leptin (ng/ml) | 21.2 (16.8) | 8.8 (8.6) | 12.3 | 127 | -9.6 | < 0.001 |
| Leptin men | 13.0 (9.7) | 5.2 (5.3) | 7.8 | 67 | -6.15 | < 0.001 |
| Leptin women | 30.3 (18.4) | 12.8 (9.8) | 17.5 | 60 | -8.15 | < 0.001 |

†: paired t-test comparison.

Abbreviations: BMI= body mass index, HOMA=homeostasis model assessment, LDL=low density lipoprotein, HDL=high density lipoprotein.

Table S3. Antipsychotic treatment at baseline, 1-year, 3-years and 10-years follow-ups.

|  | Aripiprazole  N=30 | | Risperidone  N=40 | | Olanzapine  N=40 | | Quetiapine  N=28 | | Ziprasidone  N=32 | | Haloperidol  N=39 | | Total  N=209 | | *Statistic* | *Value* | *p* |
| --- | --- | --- | --- | --- | --- | --- | --- | --- | --- | --- | --- | --- | --- | --- | --- | --- | --- |
|  | *Mean* | *SD* | *Mean* | *SD* | *Mean* | *SD* | *Mean* | *SD* | *Mean* | *SD* | *Mean* | *SD* | *Mean* | *SD* |  |  |  |
| Equivalent CPZ - Baseline | 196.7 | 18.3 | 278.8 | 64.9 | 292.5 | 69.6 | 155.7 | 63.8 | 185.2 | 63.1 | 250.8 | 83.9 | 233.6 | 81.5 | F-w | 29.329 | 0.000 |
| Equivalent CPZ - 1Y | 276.8 | 119.4 | 378.3 | 269.3 | 362.2 | 199.3 | 449.4 | 409.9 | 262.9 | 98.1 | 291.8 | 177.9 | 336.6 | 237.0 | X^2^ | 8.669 | 0.123 |
| Equivalent CPZ - 3Y | 303.0 | 197.2 | 386.8 | 239.4 | 437.5 | 388.7 | 301.9 | 254.1 | 246.8 | 135.3 | 350.0 | 206.3 | 341.8 | 255.6 | X^2^ | 10.513 | 0.062 |
| Equivalent CPZ - 10Y | 391.6 | 271.3 | 482.1 | 475.0 | 558.1 | 365.3 | 355.8 | 195.8 | 355.9 | 185.9 | 500.0 | 273.7 | 448.4 | 322.0 | X^2^ | 12.344 | 0.030 |
|  | *N* | *%* | *N* | *%* | *N* | *%* | *N* | *%* | *N* | *%* | *N* | *%* | *N* | *%* |  |  |  |
| Treatment 1Y (Yes) | 25 | 86.2 | 36 | 94.7 | 37 | 92.5 | 26 | 92.9 | 29 | 90.6 | 39 | 100.0 | 192 | 93.2 | Fisher | 5.599 | 0.253 |
| Polypharmacy 1Y (Yes) | 0 | 0.0 | 1 | 2.6 | 1 | 2.6 | 6 | 22.2 | 0 | 0.0 | 0 | 0.0 | 8 | 4.0 | Fisher | 27.917 | 0.000 |
| Main Treatment 1Y |  |  |  |  |  |  |  |  |  |  |  |  |  |  | X^2^ | 499.869 | 0.000 |
| Aripiprazole | 21 | 77.8 | 0 | 0.0 | 0 | 0.0 | 0 | 0.0 | 1 | 3.2 | 1 | 2.6 | 23 | 11.4 |  |  |  |
| Risperidone | 1 | 3.7 | 32 | 84.2 | 3 | 7.7 | 4 | 14.8 | 6 | 19.4 | 0 | 0.0 | 46 | 22.9 |  |  |  |
| Olanzapine | 0 | 0.0 | 2 | 5.3 | 29 | 74.4 | 1 | 3.7 | 2 | 6.5 | 11 | 28.2 | 45 | 22.4 |  |  |  |
| Clozapine | 1 | 3.7 | 1 | 2.6 | 0 | 0.0 | 0 | 0.0 | 1 | 3.2 | 0 | 0.0 | 3 | 1.5 |  |  |  |
| Quetiapine | 1 | 3.7 | 3 | 7.9 | 4 | 10.3 | 14 | 51.9 | 1 | 3.2 | 5 | 12.8 | 28 | 13.9 |  |  |  |
| Ziprasidone | 0 | 0.0 | 0 | 0.0 | 1 | 2.6 | 0 | 0.0 | 15 | 48.4 | 0 | 0.0 | 16 | 8.0 |  |  |  |
| Risperidone IM | 2 | 7.4 | 0 | 0.0 | 1 | 2.6 | 7 | 25.9 | 3 | 9.7 | 1 | 2.6 | 14 | 7.0 |  |  |  |
| Amisulpride | 0 | 0.0 | 0 | 0.0 | 0 | 0.0 | 1 | 3.7 | 2 | 6.5 | 0 | 0.0 | 3 | 1.5 |  |  |  |
| Haloperidol | 0 | 0.0 | 0 | 0.0 | 0 | 0.0 | 0 | 0.0 | 0 | 0.0 | 20 | 51.3 | 20 | 10.0 |  |  |  |
| Others | 0 | 0.0 | 0 | 0.0 | 1 | 2.6 | 0 | 0.0 | 0 | 0.0 | 1 | 2.6 | 2 | 1.0 |  |  |  |
| Paliperidone | 1 | 3.7 | 0 | 0.0 | 0 | 0.0 | 0 | 0.0 | 0 | 0.0 | 0 | 0.0 | 1 | 0.5 |  |  |  |
|  |  |  |  |  |  |  |  |  |  |  |  |  |  |  |  |  |  |
| Treatment 3Y (Yes) | 25 | 83.3 | 28 | 80.0 | 31 | 83.8 | 22 | 78.6 | 28 | 87.5 | 35 | 92.1 | 169 | 84.5 | Fisher | 3.236 | 0.636 |
| Polypharmacy 3Y (Yes) | 2 | 6.9 | 1 | 3.2 | 4 | 12.1 | 2 | 8.3 | 0 | 0.0 | 2 | 5.7 | 11 | 6.0 | Fisher | 4.775 | 0.439 |
| Main Treatment 3Y |  |  |  |  |  |  |  |  |  |  |  |  |  |  | X^2^ | 243.714 | 0.000 |
| Aripiprazole | 21 | 72.4 | 1 | 3.2 | 0 | 0.0 | 3 | 12.5 | 7 | 23.3 | 1 | 2.9 | 33 | 18.1 |  |  |  |
| Risperidone | 1 | 3.4 | 17 | 54.8 | 3 | 9.1 | 2 | 8.3 | 5 | 16.7 | 4 | 11.4 | 32 | 17.6 |  |  |  |
| Olanzapine | 0 | 0.0 | 3 | 9.7 | 20 | 60.6 | 1 | 4.2 | 1 | 3.3 | 10 | 28.6 | 35 | 19.2 |  |  |  |
| Clozapine | 1 | 3.4 | 2 | 6.5 | 0 | 0.0 | 3 | 12.5 | 2 | 6.7 | 0 | 0.0 | 8 | 4.4 |  |  |  |
| Quetiapine | 0 | 0.0 | 2 | 6.5 | 3 | 9.1 | 9 | 37.5 | 1 | 3.3 | 3 | 8.6 | 18 | 9.9 |  |  |  |
| Ziprasidone | 0 | 0.0 | 1 | 3.2 | 3 | 9.1 | 0 | 0.0 | 9 | 30.0 | 1 | 2.9 | 14 | 7.7 |  |  |  |
| Risperidone IM | 5 | 17.2 | 1 | 3.2 | 3 | 9.1 | 4 | 16.7 | 2 | 6.7 | 5 | 14.3 | 20 | 11.0 |  |  |  |
| Amisulpride | 0 | 0.0 | 2 | 6.5 | 0 | 0.0 | 0 | 0.0 | 0 | 0.0 | 0 | 0.0 | 2 | 1.1 |  |  |  |
| Haloperidol | 0 | 0.0 | 1 | 3.2 | 0 | 0.0 | 0 | 0.0 | 0 | 0.0 | 8 | 22.9 | 9 | 4.9 |  |  |  |
| Others | 1 | 3.4 | 1 | 3.2 | 1 | 3.0 | 2 | 8.3 | 2 | 6.7 | 3 | 8.6 | 10 | 5.5 |  |  |  |
| Paliperidone | 0 | 0.0 | 0 | 0.0 | 0 | 0.0 | 0 | 0.0 | 1 | 3.3 | 0 | 0.0 | 1 | 0.5 |  |  |  |
|  |  |  |  |  |  |  |  |  |  |  |  |  |  |  |  |  |  |
| Treatment 10Y (Yes) | 27 | 90.0 | 31 | 77.5 | 31 | 77.5 | 24 | 85.7 | 27 | 84.4 | 35 | 89.7 | 175 | 83.7 | X^2^ | 4.271 | 0.511 |
| Polypharmacy 10Y (Yes) | 6 | 22.2 | 3 | 9.4 | 8 | 25.8 | 2 | 8.3 | 2 | 7.4 | 7 | 20.0 | 28 | 15.9 | Fisher | 7.022 | 0.236 |
| Main Treatment 10Y |  |  |  |  |  |  |  |  |  |  |  |  |  |  | X^2^ | 111.865 | 0.000 |
| Aripiprazole | 14 | 51.9 | 4 | 12.5 | 4 | 12.9 | 7 | 29.2 | 4 | 14.8 | 6 | 17.1 | 39 | 22.2 |  |  |  |
| Risperidone | 0 | 0.0 | 10 | 31.3 | 2 | 6.5 | 1 | 4.2 | 2 | 7.4 | 1 | 2.9 | 16 | 9.1 |  |  |  |
| Olanzapine | 1 | 3.7 | 1 | 3.1 | 5 | 16.1 | 1 | 4.2 | 6 | 22.2 | 8 | 22.9 | 22 | 12.5 |  |  |  |
| Clozapine | 3 | 11.1 | 7 | 21.9 | 6 | 19.4 | 3 | 12.5 | 2 | 7.4 | 6 | 17.1 | 27 | 15.3 |  |  |  |
| Quetiapine | 0 | 0.0 | 3 | 9.4 | 2 | 6.5 | 3 | 12.5 | 0 | 0.0 | 2 | 5.7 | 10 | 5.7 |  |  |  |
| Ziprasidone | 0 | 0.0 | 0 | 0.0 | 1 | 3.2 | 0 | 0.0 | 4 | 14.8 | 0 | 0.0 | 5 | 2.8 |  |  |  |
| Risperidone IM | 1 | 3.7 | 2 | 6.3 | 2 | 6.5 | 0 | 0.0 | 0 | 0.0 | 3 | 8.6 | 8 | 4.5 |  |  |  |
| Amisulpride | 0 | 0.0 | 1 | 3.1 | 1 | 3.2 | 0 | 0.0 | 0 | 0.0 | 0 | 0.0 | 2 | 1.1 |  |  |  |
| Haloperidol | 0 | 0.0 | 0 | 0.0 | 0 | 0.0 | 0 | 0.0 | 0 | 0.0 | 3 | 8.6 | 3 | 1.7 |  |  |  |
| Others | 1 | 3.7 | 0 | 0.0 | 1 | 3.2 | 0 | 0.0 | 0 | 0.0 | 2 | 5.7 | 4 | 2.3 |  |  |  |
| Paliperidone | 0 | 0.0 | 1 | 3.1 | 1 | 3.2 | 1 | 4.2 | 1 | 3.7 | 1 | 2.9 | 5 | 2.8 |  |  |  |
| Paliperidone IM 1M | 5 | 18.5 | 3 | 9.4 | 3 | 9.7 | 6 | 25.0 | 3 | 11.1 | 2 | 5.7 | 22 | 12.5 |  |  |  |
| Aripiprazole IM | 2 | 7.4 | 0 | 0.0 | 3 | 9.7 | 2 | 8.3 | 5 | 18.5 | 1 | 2.9 | 13 | 7.4 |  |  |  |
